# Separating and Purifying Mycosporine-like Amino Acids from Cyanobacteria for Application in Commercial Sunscreen Formulations †

**DOI:** 10.3390/biotech12010016

**Published:** 2023-02-03

**Authors:** Valeria Candelo, Carole Anne Llewellyn

**Affiliations:** 1 Biosciences, Faculty of Science and Engineering, Swansea University, Singleton Park, Swansea SA2 8PP, UK; 2 AlgaeCytes Limited, Discovery Park, Ramsgate Road, Sandwich, Kent CT13 9ND, UK

**Keywords:** mycosporine-like amino acids, membrane filtration, sunscreens, cyanobacteria, phycocyanin

## Abstract

Using algal-derived mycosporine-like amino acids (MAAs) in sunscreen formulations is constrained by low cellular concentrations of MAAs and by the high costs associated with harvesting algal cells and extracting the MAAs. Here, we report an industrial scalable method using a membrane filtration approach to purify and concentrate aqueous extracts of MAAs. The method includes an additional biorefinery step enabling purification of phycocyanin, an established valuable natural product. Cultivated cells of the cyanobacterium *Chlorogloeopsis fritschii* (PCC 6912) were concentrated and homogenised to produce a feed for sequential processing through three membranes of decreasing pore size to obtain a retentate and permeate for each step. Microfiltration (0.2 µm) was used to remove cell debris. Ultrafiltration (10,000 Da) was used to remove large molecules and recover phycocyanin. Finally, nanofiltration (300–400 Da) was used to remove water and other small molecules. Permeate and retentate were analysed using UV-visible spectrophotometry and HPLC. The initial homogenised feed had a shinorine concentration of 5.6 ± 07 mg L^−1^. The final nanofiltered retentate resulted in a 3.3 times-purified concentrate (shinorine concentration of 18.71 ± 0.29 mg L^−1^). Significant process losses (35%) highlight scope for improvement. Results confirm the potential of membrane filtration to purify and concentrate aqueous solutions of MAAs with simultaneous separation of phycocyanin highlighting a biorefinery approach.

## 1. Introduction

Natural product replacement molecules for sunscreens are being increasingly sought due to emerging human health and environmental concerns associated with sunscreens that are synthetically produced [1]. Good candidates as replacement molecules are mycosporine-like amino acids (MAAs). MAAs are a group of around 30 small (<400 Da), colourless, water-soluble, UV-absorbing molecules found in aquatic organisms including cyanobacteria and microalgae. They have multifunctional properties including photoprotection and antioxidant activity [2,3]. Notably, they have strong UV absorption between 306–360 nm with high extinction coefficients (ε = 28,100–50,000 M cm^−1^) [4,5,6,7]. They are also photostable in fresh and saltwater even when photosensitisers are present and are resistant to abiotic stressors such as temperature and pH [7,8,9,10]. Even though their function has not yet been fully elucidated, other proposed functions include involvement in salt stress, desiccation, thermal protection and intracellular nitrogen storage [6,8,11,12]. These multifunctional properties together with their photostability and high UV absorption coefficients make them good candidate molecules as natural product replacements for synthetically produced sunscreens in skin and haircare formulations.

There are many studies and reviews on MAAs including a study showing that a hydroalcoholic formulation containing MAAs from the red alga *Porphyra umbilicalis* (Helioguard 365^®^, Mibelle industries, Buchs, Switzerland) was efficacious both in vitro and in vivo in humans [13]. Two formulations exist, both derived from red algae; the aforementioned Helioguard 365^®^ and HELIONORI^®^ by Gelyma are available to the cosmetic industry, with only a handful of MAA-containing products available to the public, often at high prices. However, the commercial availability of MAAs is still very limited. Hurdles in the commercialisation of MAAs include limited seasonal availability of some raw materials, especially seaweeds [14], and the high harvesting costs and the low cellular concentrations of alternative sources such as microalgae and cyanobacteria [15]. Furthermore, current extraction and purification methods for MAAs often require the use of solvents, as well as centrifugation, evaporation and other costly processes [2,4].

Cyanobacteria have been widely studied in biotechnology for their production of various secondary metabolites, including MAAs [16,17]. This study focuses on *Chlorogloeopsis fritschii*. *C. fritschii* is a filamentous Section V thermophilic terrestrial cyanobacterium first isolated from Indian soil [18]. It is a robust species able to withstand a wide variety of temperatures, salinities, pH and growth conditions [8,19]. It is a suitable candidate for large-scale production and biomass processing due to its ease of being maintained as a monoculture and its proven ability to grow in photobioreactors (PBR) up to 8000 l [20]. *C. fritschii* predominantly produces the MAA shinorine (SH, molecular weight: 332.306 Da, extinction coefficient: 44,700 M^−1^ cm^−1^) when exposed to UV radiation but can also accumulate SH’s precursor mycosporine-glycine (MG) [21,22]. Under UV exposure alone, *C. fritschii* has been reported to produce up to 0.15% of its dry weight of MAAs [23,24], considerably lower than the 1–1.5% of dry weight concentrations obtained by *Porphyra* sp. seaweeds, even when unexposed to UV [25]. However, *Porphyra* is already in high demand in the food industry (nori, laverbread) and can be scarce at certain times of year due to its seasonal growth. Thus, microalgae and cyanobacteria present an alternative reliable source of MAAs.

Chemical synthesis of MAAs has proven difficult [26], and heterologous production using bacteria and yeast is still developing and not available for commercial production [27,28]. Therefore, novel techniques like membrane filtration are a solution to extracting MAAs from readily available sources at a low cost and could help fill the gap in the market in developing natural sunscreen formulations.

Membrane filtration techniques have been utilised in various aspects of biotechnology, including harvesting of microalgae and cyanobacteria [29,30,31] and purification of protein and pigments for the food and cosmetic industries [13,32,33,34]. Some of the advantages of membrane filtration include rapid and efficient processing of products, lower operational costs and higher quality compared to other purification methods [35]. Membrane filters also offer the benefit of separation at room temperature and enhanced resistance to microbial and chemical degradation compared to other filtration methods. Furthermore, the ability to process tens of thousands of cubic meters of products a day has made membrane filtration a well-established industrial and large-scale process [36]. Membrane filtration processes are classified by the driving force of the separation (i.e., pressure, concentration) and the size of the materials to be separated [36]. Membrane filtration has been shown to have great potential in concentrating and purifying other high-value compounds in cyanobacteria’s crude extracts such as the blue pigment phycocyanin (PC) [37]. However, membrane processing has not yet been applied to processing aqueous extracts of MAAs.

The overall aim of this study was to develop a new commercially scalable membrane filtration processing method to concentrate and purify MAAs from aqueous extracts of cyanobacteria working towards a biorefinery approach with parallel separation of phycocyanin. The suitability of *C. fritschii* for large-scale production was tested by culturing the biomass used as the feed in an 800 l photobioreactor. The feed was then processed through three-step sequential filtration using microfiltration (0.3 µm; MF), ultrafiltration (10,000 Da; UF) and nanofiltration (300–400 Da; NF) membranes.

MF was used to remove cell debris, UF was used to remove large molecules and recover phycocyanin, and NF was used to remove water and other small molecules. Permeate and retentate were analysed using UV-visible spectrophotometry and high-performance liquid chromatography (HPLC), the latter of which was set up to measure MAAs. A quantitative mass balance was performed to determine losses.

## 2. Materials and Methods

### 2.1. Culture Growth

*Chlorogloeopsis fritschii* PCC 6912, originally purchased from the Pasteur Culture Collection, was obtained as a master culture from the Centre for Sustainable Aquaculture (CSAR), Swansea University. It was first upscaled in BG 11 medium and then acclimatised and grown in the pilot scale PBR using F/2 (CellHi F2P, Varicon Aqua) as growth medium. The culture was grown during July 2019 in an 800 L horizontal tube PBR (BioFenceTM, Varicon Aqua, UK) in the greenhouse facility at Swansea University Singleton Campus. Temperature was maintained around 25 °C by a cooling water sprinkler system and pH maintained around 8 with automated solenoid valve CO_2_ injections. The average pH of the culture was 8.25 ± 0.99. High pH variation was due to the malfunctioning of the pH regulation system that required correction with sodium bicarbonate and CO_2_ injections. The average daily PBR temperature was 25.3 ± 1.8 °C. The average daily light intensity varied greatly depending on the weather conditions, ranging from 313.5 to 4173.2 μmol m^−2^ s^−1^. Culture density was monitored as dry weight every other day. Measurements were taken in duplicates using pre-dried (24 h at 80 °C) and pre-weighed GF/C glass fibre filters (Whatman). A known volume of culture (1, 5 or 10 mL, depending on culture density to avoid filter clogging) was filtered using a Millipore filtration system, rinsed with DI water and dried in an oven (80 °C) for at least 24 h. The difference in filter weight was used to calculate the dry weight concentration using the following Equation:

DW= (W*d* − W*f*)/*v* (1)

where:

DW: concentration in g L^−1^ of dry weight

W*d*: weight of the dried filter + biomass

W*f*: weight of the dry clean filter

*v*: volume in litres of culture filtered

### 2.2. Biomass Preparation

Ten l of culture were harvested from the stationary growth phase at 34 days and replaced with fresh media. The sample was then concentrated to 3 l using membrane microfiltration, and then to 50 mL with a combination of sedimentation and centrifugation. The biomass was stored frozen at −20 °C until further processing. Once thawed, the biomass was homogenised using a cell disruptor (Constant systems, Daventry, UK) operated at 30,000 psi to create the feed for the filtration experiment. Disruption efficiency was calculated as the percentage difference in intact cells counted before and after disruption. This was obtained by counting the number of intact cells in 100 μL of processed and unprocessed sample using a haemocytometer.

### 2.3. Membrane Filtration

**Figure 1:**
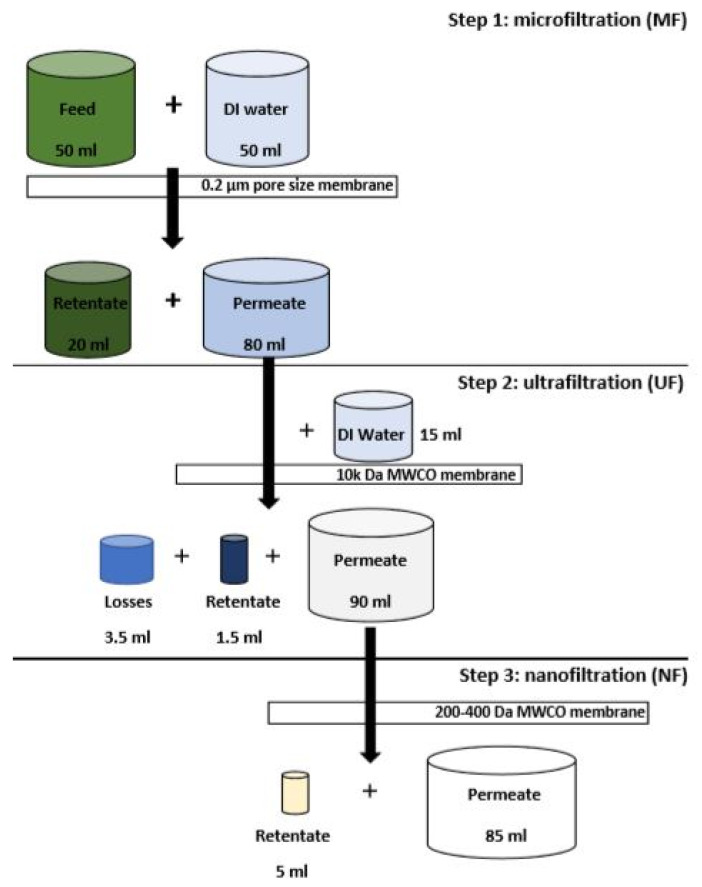
Schematic of the sequential three-stage membrane filtration process.

A schematic of the filtration process is shown in Figure 1. Fifty mL of feed was processed using a 100 mL dead-end low pressure stirred filtration cell (Merck Amicon) fitted with an MF flat sheet membrane (Sterlitech YMJXSP3001, PVDF, pore size: 0.3 μm) pre-soaked in DI water operated at 2.4 bar. A further 50 mL of DI water was added in batches throughout the process to dislodge caked debris. Eighty mL MF permeate (MFP) was then filtered in the same filtration cell fitted with a flat sheet UF membrane (Microdyn-Nadir, 10,000 Da MWCO) pre-soaked in DI water and operated at 2.4 bar. Towards the end of the filtration, 15 mL of DI water were used to rinse UF retentate (UFR) residues from the filtration cell. Finally, the 90 mL of UF permeate was filtered in a 200 mL metal high pressure stirred filtration cell and a flat sheet NF membrane (Filmtec membranes, NF270, ~200–400 Da MWCO) pre-soaked in DI water operated at 15 bar.

### 2.4. Sample Preparation and Analysis

Samples of both permeate and retentate were collected after each filtration step and centrifuged to remove solids when present. They were then stored at −80 °C until further analysis.

#### 2.4.1. Spectrophotometry

All spectrophotometry measurements were taken using a Spectrostar nano spectrophotometer (BMG Labtech). Absorption spectra (280–800 nm) were recorded using 1 cm quartz cuvettes using DI water as blank. Samples were diluted to bring OD measurements within the linear range of 3.5 and then multiplied by the appropriate dilution factor.

To estimate the PC concentration in the fractions, the OD at 592 nm, 618 nm and 645 nm was extrapolated from the recorded spectra and applied to the following equation from Beer and Eshel [38]:

PC (mg mL^−1^) = [(OD618 nm − OD645 nm) − (OD592 nm − OD645 nm) × 0.15] × 0.15 (2)

#### 2.4.2. High Performance Liquid Chromatography (HPLC) Analysis

The MAA content in the feed, and in the retentate and permeate of each filtration step, was analysed using an HP Agilent 1100 HPLC system using a 5 μm, C-18 column (Alltima™ Altech™, 120 Å pore size, 150 mm × 4.6 mm) as the stationary phase operated at 35 °C. This method was optimised for MAA separation by Kultschar et al. [39]. All samples were analysed in triplicates (technical replicates) with an injection volume of 50 μL. Peaks were detected with monitoring at 330 nm using a Diode Array Detector and absorption spectra recorded for each peak between 200–400 nm. In the absence of standards, SH was identified using a combination of retention time and peak maximum absorbance (λmax). The feed and the MFR fractions were very dense and rich in PC, namely, and were diluted accordingly by up to 40 times to avoid overloading of the HPLC column.

The total peak area (TPA) of SH in each filtration fraction was calculated by normalising the average peak area of the sample for the volume of the fraction obtained using the following equation:

TPA = Pa*s* × V*f*/V*s* (3)

where:

Pa*s* = Average peak area of the sample

V*f*: volume of the fraction (mL)

V*s*: volume of the sample (0.05 mL)

An estimate of the SH concentration was calculated using the following equation adapted from the Beer–Lambert law:

c = A/εl × MW(4)

where:

c: concentration (mg L^−1^)

A: average absorbance (mAU)

ε: extinction coefficient (M^−1^ cm^−1^)

l: path length (mAU readings are normalised to a path length of 1 cm)

MW: molecular weight (g mol^−1^)

The percentage of SH retained by the membrane, or retention coefficient (R%), was determined after each filtration using the following equation adapted from Richardson et al. [36]:

R% = (C*f* − C*p*)/C*f* × 100 (5)

where:

C*f*: SH average peak area in the feed stream

C*p*: SH average peak area of the permeate.

#### 2.4.3. Mass Balance Calculations

To calculate the mass balance of the filtration, average TPA of SH from the HPLC analysis was used, as peak area is directly proportional to mass. The fraction’s TPA was converted to the percentage of the TPA of the feed using the following equation:

TPA% = TPA*f*/TPA*Feed* × 100 (6)

where:

TPA*f*: total average peak area of the fraction

TPA*Feed*: total average peak area of the feed

Processing losses for each filtration step were quantified as the difference between the starting TPA% before filtration and the sum of the TPA% measured in the obtained fractions. The mass balance for SH for the entire filtration process was obtained as the sum of % of SH recovered in the target fraction (NFR), the SH lost in the non-target fractions (MFR, UFR, NFP) and the processing losses in each filtration.

## 3. Results and Discussion

### 3.1. Culture Growth

**Figure 2:**
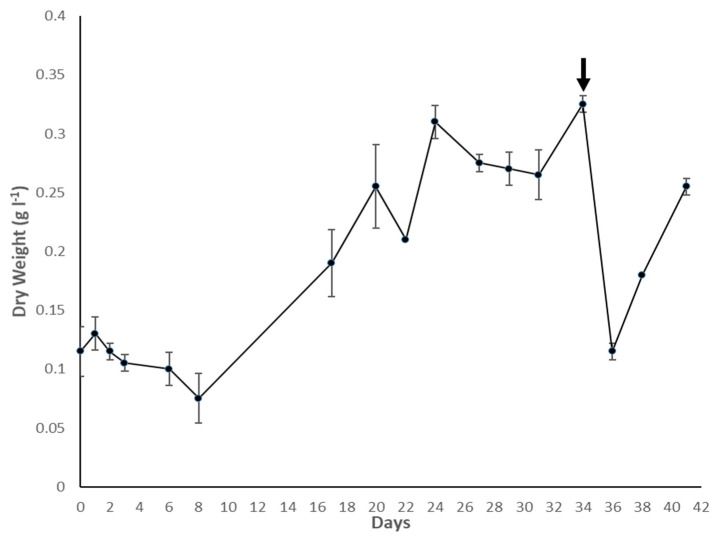
Growth curve as dry weight of *C. fritschii* in the PBR. Error bars represent standard deviation. The black arrow indicates the time point at which the biomass was harvested.

The density (g L^−1^ of dry weight) of the culture throughout the growth period of 41 days is depicted in Figure 2. A few technical difficulties with the CO_2_ supply coupled with unfavourable weather resulted in variable and suboptimal growth conditions. Despite this, the culture grew steadily, although at a slower rate than that observed during the initial scale-up process. This confirmed the robustness and resilience of *C. fritschii* to variable growth conditions typical of large-scale production such as outdoor raceway ponds. However, right after the harvest of 10 l for the filtration experiment, the culture density dropped to 0.12 g L^−1^ after 36 days, possibly due to the combination of lower biomass density due to the harvest and particularly high temperatures experienced in the days following the harvest. The culture density increased back to 0.25 g L^−1^ by day 41; however, it was found to be caused by contamination by the green microalga *Scenedesmus quadricauda*, grown in a separate reactor in the same greenhouse. Despite this, no traces of the contaminant were found in the experimental samples. The final biomass density reached at harvest was of 0.32 g L^−1^, obtaining 50 mL of concentrated homogenised feed of 24 g L^−1^ of dry weight.

### 3.2. Filtration Experiment

The cell disruptor processed the 50 mL concentrated sample with a 98% efficiency in less than a minute with only one pass. The use of wet biomass and aqueous extraction has been shown to be more effective in extracting MAAs than solvent-extracted dry biomass while requiring less energy and preparation [4]. Although cell disruptors have shown potential in the processing scale, a few limitations call for the investigation of other suitable homogenisation techniques best suited at large scale [20,40,41]. For example, pilot-scale ball mills have been shown to successfully disrupt large quantities of PBR-grown, *C. fritschii* wet biomass with low energy expenditures [20].

#### 3.2.1. Phycocyanin

**Figure 3:**
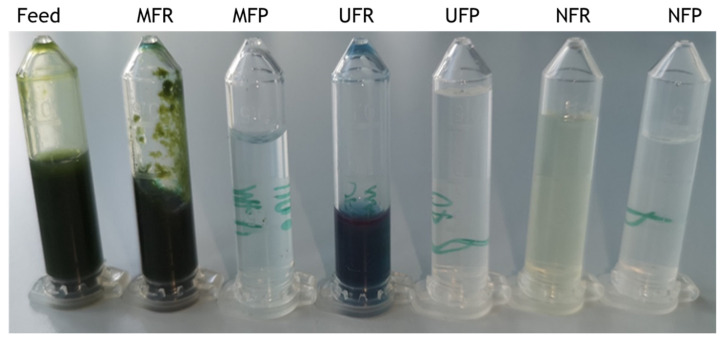
Different fractions obtained during the three membrane filtrations. From left to right: *C. fritschii* feed solution, microfiltration retentate (MFR), microfiltration permeate (MFP), ultrafiltration retentate (UFR), ultrafiltration permeate, (UFP), nanofiltration retentate (NFR) and nanofiltration permeate (NFP).

During preliminary investigations, a high quantity of phycocyanin (PC) was observed in the feed, suggesting a possible biorefinery approach to this method for the recovery of multiple bioproducts. However, cell debris hindered UF and fouled a PC-rich extract. This led to the investigation of MF to remove cell debris before UF. This was motivated by MF’s low operational cost compared to centrifugation and its scalability potential, similar to the other membranes used [29,42]. Photos of samples from all the filtration fractions are shown in Figure 3 MF yielded a light blue, debris-free MFP and a thick, green slurry as MFR. A high PC concentration was found in the MFR (0.87 mg mL^−1^), as significant caking was observed during filtration. Caking is a form of membrane fouling in which solids are strained and accumulated on the surface of the membrane [43]. Caking reduces filtration flow rate and efficiency and can influence the actual MWCO of the membranes, as small molecules are retained by the cake of larger particles on the surface [30,34,43]. Protein and polysaccharide substances are two of the major membrane foulants [30,44]. Although the washing of the biomass in DI water aimed to remove external polysaccharides, proteins and other cell constituents were released in the feed with homogenisation. Caking reduced the permeability of phycocyanin observable in a change of colour of the permeate, suggesting the retainment of PC, that with a MW of 264,000 Da should easily permeate the 0.3 μm MF membrane pore size [45]. Small amounts of DI water were used to dislodge the cake layer to restore PC permeability, but resulted in the interruption of the filtration process several times. The filtration was stopped once the retentate became a thick slurry and the flow of permeate came to a stop.

**Figure 4:**
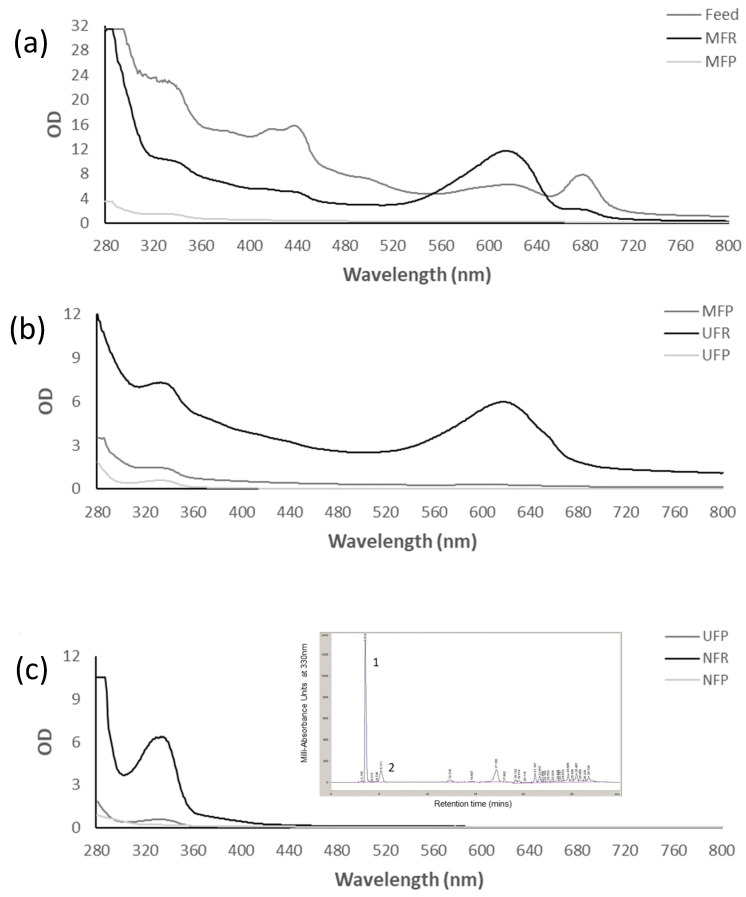
Absorption spectra of MF (**a**), UF (**b**) and NF (**c**) fractions with inset showing the corresponding NFR HPLC chromatogram with peaks 1 and 2 identified as shinorine and mycosporine-glycine, respectively. OD numbers are adjusted to reflect dilutions.

From spectrophotometric data (Figure 4), the initial PC concentration in the feed (50 mL) was calculated as 0.23 mg mL^−1^, 0.87 mg mL^−1^ in the MFR (20 mL), and only 0.007 mg mL^−1^ in the MFP (80 mL), suggesting retention of PC by the MF membrane, as shown by the change in colour of the MFP during filtration due to caking. However, UF was able to successfully reconcentrate the PC to 0.23 mg mL^−1^ in the UFR (1.5 mL), with <0.0001 mg mL^−1^ of PC left in the UFP (90 mL). Potential improvements to the process including cross-flow filtration and continuous diafiltration should be investigated to improve the MAA and PC yield of the process by reducing losses in non-target fractions [29,43].

#### 3.2.2. MAAs

For the MAAs, the retention coefficient for SH during MF was 18%, of which 11% was retained in the MFR, while 7% was lost through processing losses (Table 1). Although 82% of MAAs present in the feed passed through in the MFP, the substantial loss can be attributed once again to caking and the filtration apparatus. The use of batch diafiltration was implemented to aid MAAs permeation and avoid caking. Diafiltration consists of the addition of a diafiltration liquid to help ‘wash away’ low MW particles from a retentate [46]. In this study, DI water was used as the diafiltration liquid and added in batches to the filtration cell to dislodge the cake layer and dilute the slurry-like feed to reduce caking. This partially aided the filtration flow rate and PC permeation; however, it required the interruption of the process, making it time- and labour-consuming. This was feasible at the bench scale working with small volumes, but it would be challenging to apply at a larger scale.

A UF membrane was selected guided by evidence in the literature of the use of 10,000 Da MWCO membranes for the processing of MAAs and pigments in seaweed extracts [13,35]. UF resulted in a debris-free deep blue retentate and a clear permeate; however, the yield of the UFR was reduced by its adherence to the filtration chamber, and 3.5 mL were lost. NF yielded a light yellow NFR and a clear NFP. 

The absorption spectra of the fractions (Figure 4) were used to predict concentrations of MAAs in the different fractions before HPLC analysis and to investigate changes in the composition of the fractions throughout filtration. After MF (Figure 4a), the MFR absorbance in the MAA region was reduced compared to the feed, but still quite prominent. The MFP showed some absorbance at the 280–360 nm wavelengths, but minimal absorbance at other wavelengths, suggesting permeation of MAAs and the removal of cell debris and other pigments by the MF membrane. After UF (Figure 4b), the MAAs absorbance in the UFR increased to about five times the MFP levels, while MAA absorbance in the UFP was low. NF (Figure 4c) was very effective in concentrating the low concentration of MAAs present in the UFP, achieving 10-fold concentrations in the OD in the MAA region.

**Figure 5:**
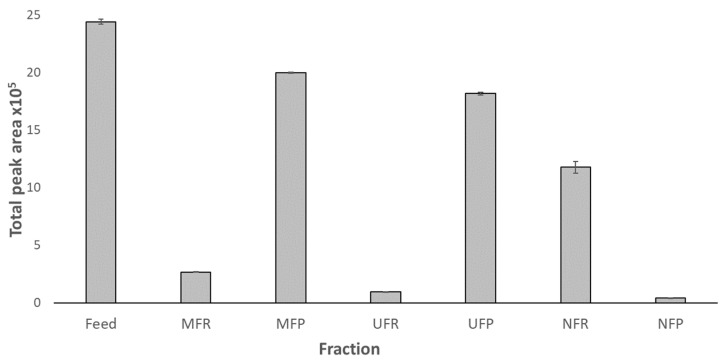
Total peak area of shinorine (SH) for each fraction calculated from HPLC data for the feed, and membrane filtration permeates and retentates. Peak area normalised for the volume of each fraction obtained during filtration. Error bars represent standard deviation.

The presence of MAAs in the feed suggested by the absorption spectra was confirmed by HPLC analysis (Figure 5). The MAA concentration in non-target retentates increased, as seen by the increase in OD in the spectra (Figure 4) and the calculated concentrations from mAU obtained from HPLC (Table 2). However, when related to the small volume obtained in these fractions, the majority of MAAs were present in the permeates, but at lower concentrations. Although between 6 and 18% of MAAs were lost during MF and UF, it was confirmed that the NF membrane could successfully concentrate MAAs retaining >97% of SH. The process resulted in 5 mL of NFR containing 18.71 ± 0.29 mg L^−1^ of purified SH.

**Figure 6:**
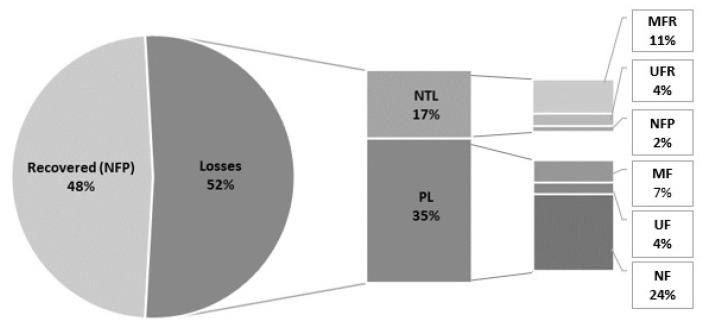
Mass balance of SH in the three-step filtration process as % of the feed. NTL: Non-target fraction losses (MFR, UFR, NFP); PL: processing losses (in MF, UF and NF).

However, the retention coefficients presented in Table 1 do not take in consideration losses incurred during filtration; therefore, a mass balance calculation is presented in Figure 6. Overall, our results showed 48% of the MAAs in the feed being recovered in a final purified extract (Figure 6). Losses of 17 and 35% were incurred, respectively, in both the non-target fractions, i.e., in the MFR, UFR, and NFP and during processing, highlighting the scope for method improvement (Figure 6).

## 4. Conclusions

We have developed a novel bioprocess using sequential membranes to separate and concentrate mycosporine-like amino acids (MAAs) from aqueous extracts of the cyanobacterium *Chlorogloeopsis fritschii*. We observed elevated processing losses highlighting scope for improvement. Purifying and concentrating MAAs is currently a bottleneck to the commercial development of MAAs in sunscreen formulations. The bioprocess we developed for MAAs can simultaneously separate phycocyanin, highlighting the potential of a biorefinery approach to separating and purifying industrially sought natural products.

## Figures and Tables

**Table 1:** Retention coefficients calculated from detected, normalised SH peak areas.

Filtration	Retention Coefficient (% ±SD)
MF	18.1 ± 0.7
UF	9.1 ± 0.5
NF	97.6 ± 0

**Table 2:** Volume and MAAs concentration of all filtration fractions calculated from mAU.

Fraction	Volume (mL)	Concentration (mg L^−1^ ± SD)
Feed	50	5.6 ± 0.7
MFR	20	1.2 ± 0.04
MFP	80	1.9 ± 0.14
UFR	1.5	5.3 ± 0.61
UFP	90	1.2 ± 0.09
NFR	5	18.71 ± 0.29
NFP	85	0.007 ± 0.004

## Data Availability

Data is available through contact with the authors.
